# The endocrine pancreas during exercise in people with and without type 1 diabetes: Beyond the beta-cell

**DOI:** 10.3389/fendo.2022.981723

**Published:** 2022-09-06

**Authors:** Olivia McCarthy, Signe Schmidt, Merete Bechmann Christensen, Stephen C. Bain, Kirsten Nørgaard, Richard Bracken

**Affiliations:** ^1^ Applied Sport, Technology, Exercise and Medicine Research Centre, Swansea University, Swansea, United Kingdom; ^2^ Steno Diabetes Center Copenhagen, Copenhagen University Hospital, Herlev, Denmark; ^3^ Medical School, Swansea University, Swansea, United Kingdom; ^4^ Department of Clinical Medicine, University of Copenhagen, Copenhagen, Denmark

**Keywords:** type 1 diabetes, exercise, pancreatic hormones, endocrine pancreas, physical activity

## Abstract

Although important for digestion and metabolism in repose, the healthy endocrine pancreas also plays a key role in facilitating energy transduction around physical exercise. During exercise, decrements in pancreatic β-cell mediated insulin release opposed by increments in α-cell glucagon secretion stand chief among the hierarchy of glucose-counterregulatory responses to decreasing plasma glucose levels. As a control hub for several major glucose regulatory hormones, the endogenous pancreas is therefore essential in ensuring glucose homeostasis. Type 1 diabetes (T1D) is pathophysiological condition characterised by a destruction of pancreatic β-cells resulting in pronounced aberrations in glucose control. Yet *beyond the beta-cell* perhaps less considered is the impact of T1D on all other pancreatic endocrine cell responses during exercise and whether they differ to those observed in healthy man. For physicians, understanding how the endocrine pancreas responds to exercise in people with and without T1D may serve as a useful model from which to identify whether there are clinically relevant adaptations that need consideration for glycaemic management. From a physiological perspective, delineating differences or indeed similarities in such responses may help inform appropriate exercise test interpretation and subsequent program prescription. With more complex advances in automated insulin delivery (AID) systems and emerging data on exercise algorithms, a timely update is warranted in our understanding of the endogenous endocrine pancreatic responses to physical exercise in people with and without T1D. By placing our focus here, we may be able to offer a nexus of better understanding between the clinical and engineering importance of AIDs requirements during physical exercise.

## Introduction

Regular exercise conveys multiple health benefits for people with type 1 diabetes (T1D) including improvements in both physical and psychological wellbeing (1, 2). Nevertheless, exercise is a physiological and metabolic stressor that acutely causes considerable glycaemic disturbance; the correction of which is normally under strict homeostatic control.

In a healthy individual, the preservation of blood glucose within a relatively narrow physiological range (between 4.0 and 10.0 mmol.L^-1^) is governed by a complex array of endogenous feedback and feedforward mechanisms. Even the smallest oscillations in blood glucose initiate a continuous communicative interplay between multiple organ systems that function to balance the rate of glucose appearance (R*_a_* glucose) to that of its disappearance (R*_d_* glucose) in working muscle tissue (3).

**Figure 1:**
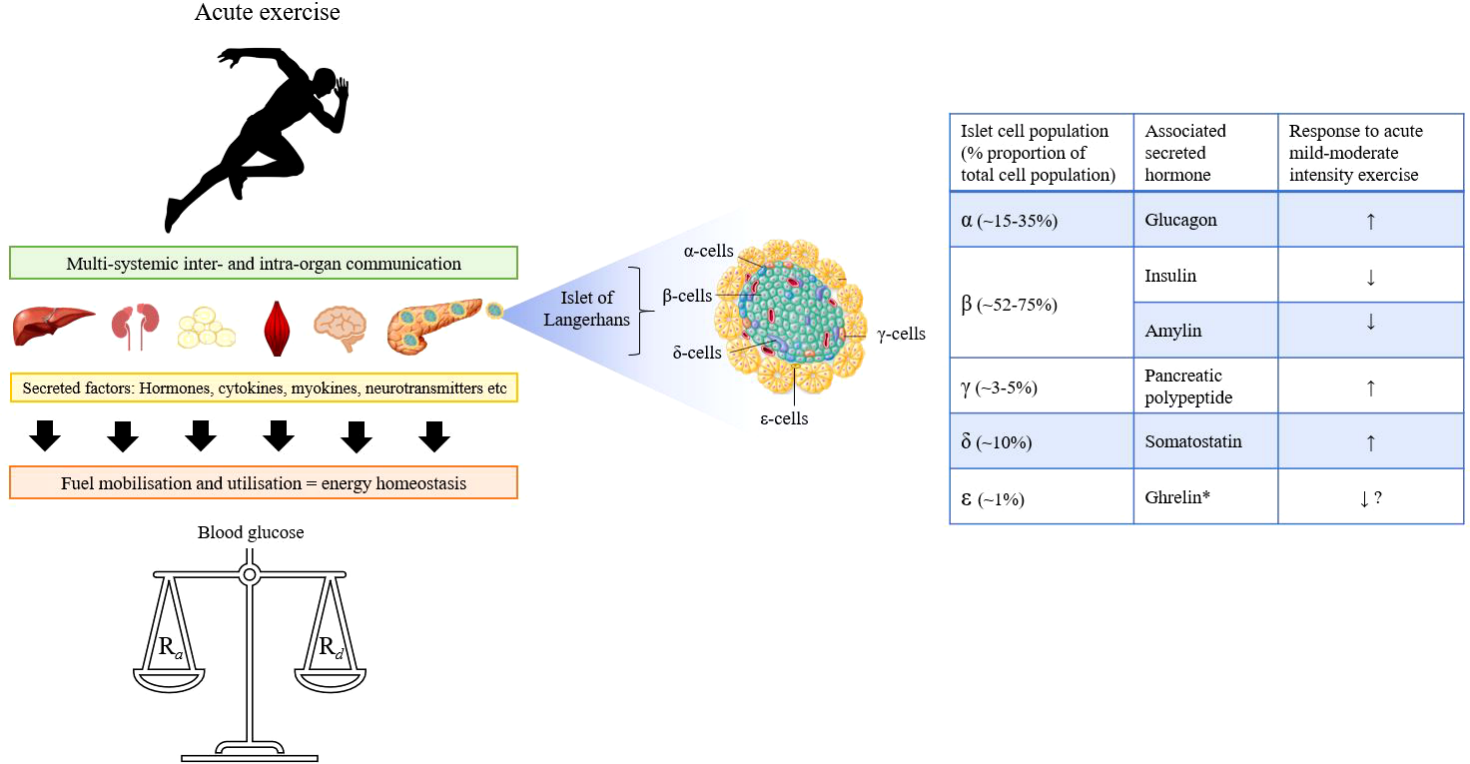
Graphical overview of the multi-systemic effects of exercise involving communication across multiple organs to ensure energy homeostasis and regulation of blood glucose in healthy persons. Blood glucose is a dynamic state of constant turnover and multiple feedforward and feedback mechanisms operate to balance the rate of glucose appearance (R_a_) with its disappearance (R_d_) during exercise. Central to this process is the pancreas, which contains five major islet-cell populations each of which secrete their associated hormone(s) depending on the metabolic requirements. *Note: Ghrelin is only produced to a very limited extent in the pancreas.

Central to this process, is the pancreas, which acts as a ‘control hub’ for several major glucoregulatory hormones (**Figure 1**). Endogenous pancreatic β-cell insulin suppression alongside increased α-cell glucagon secretion lead a hierarchy of glucoregulatory hormonal responses that help maintain blood glucose concentrations within tight limits and supply fuel to working tissue (4–6). Additional support from the sympathoadrenal system facilitates continued energy provision in the face of increasing intramuscular glucose demands (7, 8).

Of the islet-cell populations within the pancreas, the β-cells occupy the largest portion, and are considered sensory controllers that continually adjust the rate of insulin secretion to regulate systemic glucose concentrations (9, 10) Hence, their pathophysiologic destruction as a result of T1D impairs innate glucose counter-regulation and necessitates a dependency on exogenous insulin. However, exogenous insulin is not subject to endogenous feedback control, and total peripheral insulin concentrations may rise over exercise due to an increased mobilisation from the subcutaneous depot and a reduction in clearance (11). Pancreatic α-cell glucagon and sympathoadrenal system responses to exercise are also often attenuated in T1D, especially in those with long standing disease (12). These counter-regulatory hormonal deficits place a considerable reliance on diligent adherence to alterations in exogenous insulin therapy to support glycaemia around exercise (2).

Rapid advances in the field of diabetes technology have led to the development of automated insulin delivery systems (AIDs), which combine an insulin pump and a dosing algorithm that dynamically controls the insulin infusion rate based on continuous glucose monitoring. Research has demonstrated superiority in clinical outcomes (e.g., reductions in hyper- and hypo-glycaemia) when using AIDs compared to conventional insulin pump therapy (13). Despite considerable progress having been made, fundamental issues in pump performance around exercise remain apparent including overt hyperinsulinaemia and resultant hypoglycaemia. Hence, notwithstanding its value in improving many indices of human health, exercise continues to represent an *‘Achilles’ heel’* in diabetes care.

Consensus guidelines for optimal blood glucose management around physical activity in people with T1D using the current generation of diabetes therapies exist (14). The majority of these guidelines are based on delineating the metabolic responses that occur to exercise under varying degrees of exogenous insulin therapy adjustment. Yet, the responses of all other pancreatic endocrine islet-cell hormones and whether they contribute to orchestrating the liberation and utilisation of metabolic substrates during exercise is less well known, particularly in pathological states where pancreatic function is compromised i.e., T1D.

This review considers further the endocrine pancreas’ role in modulating glucose regulation during physical exercise in those with and without T1D. Though the endocrine pancreas is also instrumental in facilitating the homeostatic control of other substrates (i.e., lipids and proteins), the current topic takes a glucocentric approach given the major clinical issues faced by those with T1D. Understanding the physiology and potential pathophysiology of pancreatic cells is warranted to fully appreciate how the pancreas strives to achieve good metabolic control under states of metabolic stress. It is hoped that AID developers may appreciate the subtleties of endocrine pancreatic responses to stimuli such as exercise in their pursuit of better ‘next generation’ devices for patients with T1D.

Undoubtedly, the ask of any exogenous system to mimic such a highly sophisticated endogenous one is an understandably challenging task that is justifiably complex. Nevertheless, as we continue to develop new diabetes technologies, it is important we recognise exercise as a factor for consideration.

## The pancreas: Basic physiology and function

The pancreas is both an exocrine and endocrine gland that is heavily involved in the digestion and assimilation of nutrients through the production and secretion of various enzymes. For an understanding of the functional exocrine role and the crosstalk between pancreatic compartments, readers are directed to alternative sources for further information (15–18). The normal endocrine pancreas contains 1-3 million pancreatic islets comprised of five types of endocrine/paracrine cells (**Figure 1**) that secrete the hormones: glucagon (~15-35%, α-cells), insulin and amylin (~52-75%, β-cells), pancreatic polypeptide ([PP] ~3-5%, γ-cells), somatostatin (~10%, δ-cells), and ghrelin (~1%, ϵ-cells) (19). Worth noting is that the relative proportion of each population varies considerably depending on factors such as islet size, age and region within the organ (20–24). The secretion and suppression of these peptide hormones through intra-organ communication is essential in regulating intermediary metabolism (25). The role of the pancreas in nutritive digestion and assimilation is well cited in the literature and reviewed elsewhere (26). Yet *beyond the beta-cell*, far less is known about the wider pancreatic endocrine cell responses to dynamic physical exercise.

## The natural pancreas response to exercise

### Pancreatic blood flow during exercise

Blood is fed to the pancreas by the splenic, mesenteric (inferior and superior) and common hepatic arteries and drained by the splenic, mesenteric, and hepatic portal veins (20, 27). At rest, splanchnic organs (intestines, pancreas, spleen, and liver) receive a fifth of resting cardiac output (~1 L.min^-1^) with 20-30% of blood volume stored in abdominal veins. This ‘resting blood sump’ is diverted towards working muscle vasculature during exercise and is modulated by sympathoadrenal system influences on vasculature, and by metabolic and vasoactive peptides (e.g., vasoactive intestinal peptide, atrial natriuretic peptide, glucagon) competing against vasopressin and angiotensin. Undertaking sustained sub-maximal exercise increases cardiac output to more than 20 L.min^-1^ in man and systemic adjustments reduce blood flow rate around splanchnic organs by up to 95% of exercising cardiac output. However, overall, blood flow need for splanchnic areas is largely maintained (i.e., rest: 20% of 5 L.min^-1^ = 1 L.min^-1^ and exercise: 5% of 20 L.min^-1^ = 1 L.min^-1^). The degree of reduction in splanchnic blood flow with exercise is influenced by exercise intensity and altered in magnitude by body position, heat, aging and training state. For example, heavy upright exercise can readily reduce resting splanchnic blood flow by 70-80% and to levels that might indicate splanchnic ischaemia (28, 29).

### The influence of exercise on the release of pancreatic hormones

This section summarises the available literature detailing the influence of physical exercise on the release of pancreatic hormones *via* a cell-by-cell approach in the absence and presence of T1D.

#### Insulin (β-cell)

##### Healthy state

Physical exercise increases glucose uptake *via* muscular contraction mediated mechanisms independent of, but in addition to the action of insulin. As such, a reduction in circulating insulin concentration is necessary during exercise to avoid a disproportionate increase in the rate of glucose uptake (6). The magnitude of insulin reduction is related to the exercise characteristics that is, the work intensity and/or its duration. When workload exceeds ~50% $\dot{\text{V}}{\text{O}}_{2\text{max}}$ there is a concurrent reduction in peripheral plasma insulin concentrations. This decline is not thought to be due to blood flow redistribution or liver clearance, as C-peptide (which is co-released with insulin from the pancreas) is poorly cleared by the liver yet shows similar responses to exercise (30). Rather, the exercise-induced increase in insulin clearance observed in both healthy [+9%] and insulin-dependent [+15%] persons (31), is primarily driven by an increase in insulin degrading enzyme expression and activity in skeletal muscle (32). This may be due to feedback mechanisms involving inter-organ cross talk between skeletal muscle and the pancreas and/or neural modulation including activation of the sympathetic nervous system (33). Furthermore, the magnitude of reduction in insulin secretion is more marked in conditions of increased sympathetic nerve stress such as low cardiorespiratory fitness or low O_2_ availability. Interestingly, plasma adrenaline does not appear to exhibit the same relationship as noradrenaline, pointing towards α-adrenergic nerve fibre control of pancreatic α-cells rather than adrenal medulla chromaffin cell adrenaline release influencing adrenergic β-cells on the pancreatic cells. Though glucose should always be considered in the context of insulin dynamics, insulin concentrations may still decrease in the absence of a reciprocal fall in plasma glucose and equal reductions in insulin have been shown where blood glucose concentrations have been allowed to decrease or kept euglycaemic. Restoration of glucose *via* infusion during the final 15 minutes of exhaustive exercise did not result in a rise in insulin (30). On cessation of exercise, plasma insulin concentrations have been shown to increase in line with reduced sympathetic nerve activity and the transient rise blood glucose concentrations. There is a dual shift in lower insulin secretion and heightened insulin clearance for at least 12-44 hours after exercise, the effect of which is observed in both healthy persons and individuals with insulin-dependent diabetes (31). The physiologic aim of which may be to enhance lipolysis and hence, spare glucose utilisation under conditions of possible glycogen depletion (34) .

##### Type 1 diabetes

Though conventionally considered a condition of absolute insulin deficiency, it is now recognised that a large portion of individuals with long-standing T1D retain signs of pancreatic β-cell activity, as quantified *via* C-peptide status (35). Unlike insulin, there is negligible hepatic extraction of C-peptide, hence its value as a potentially better indicator of endogenous insulin secretion. Whilst our understanding of total (endogenous and exogenous) plasma insulin changes during exercise in T1D has been characterised, remarkably, fewer studies have explored the *endogenous* pancreatic β-cell responses. Early work by Heding and Ludvigsson (36), demonstrated that in newly diagnosed (3 months) T1D children with significant residual β function (fasting C-peptide values of 0.13-0.53 pmol.mL^-1^), plasma C-peptide and proinsulin fell over exercise (20 mins stationary cycling) but unlike in those without diabetes, failed to increase upon cessation. Though no between-group comparisons were made, the magnitude of the drop in C-peptide and proinsulin was notably lower in those with T1D (36). We recently investigated metabolic and neuroendocrine hormonal responses to exercise (45 mins cycling at ~60% V.O_2max_) and hypoglycaemia in people with T1D when stratified based on their level of residual pancreatic β-cell function (37). Participants with stimulated (1 hour post prandial) C-peptide values of ≥30.0 pmol.L^-1^ were considered low level secretors (LLS) and those with <30.0 pmol/L were classified as micro-secretors (MS). LLS had a greater suppressive effect on both C-peptide (LLS: -60.9 ± 50.5 vs MS: -0.6 ± 8.8 pmol.L^-1^, p<0,001) and proinsulin (LLS: -1.5 ± 2.6 vs MS: -0.1 ± 1.1 pmol.L^-1^, p=0.01) as well as greater adrenaline responses to exercise. Furthermore, during exercise *superimposed* with acute mild hypoglycaemia (blood glucose ≤3.9 mmol.L^-1^), LLS presented with higher sympathoadrenal (adrenaline and noradrenaline), and pancreatic β-cell (C-peptide and proinsulin) biomarker concentrations than MS (37). It was also noted that LLS spent significantly less time with interstitial glucose (*iG*) values in the hypoglycaemic range (<3.9 mmol.L^-1^) over the early (~6 hours) post-exercise period and had considerably lower glycaemic variability (CoV and SD) throughout the nocturnal hours. Complementary work by Taylor et al. (38), documented that compared to patients with low (10-190 pmol.L^-1^) and undetectable (<10 pmol.L^-1^) values of C-peptide, those with the highest levels (≥200 pmol.L^-1^) spent more time with *iG* values in range (4-10 mmol.L^-1^), less time above range (>10 mmol.L^-1^), and had lower standard deviation in *iG* in the 12 and 24 hours following acute exercise. Taken collectively, it appears that the degree of residual pancreatic β-cell function corresponds favourably with glycaemic outcomes both during and after moderate intensity continuous exercise in people with T1D. From a clinical perspective, these data may imply a potentially higher risk of hypoglycaemia during exercise in patients with advanced pancreatic β-cell loss.

#### Amylin (β-cell)

##### Healthy state

Amylin is co-secreted in equimolar quantities alongside insulin from the pancreatic β-cells. Amylin’s mediatory role in facilitating glucose homeostasis has been established *via* multiple mechanisms: (i) suppression of endogenous, post-prandial, glucagon production; (ii) lessening gastric emptying time; (iii) centrally mediated induction of satiety (39, 40). Appreciating the relationship between energy metabolism and exercise that involves amylin, Kraemer and colleagues (41) investigated amylin responses to progressive intermittent exercise protocol ranging from moderate (60% $\dot{\text{V}}{\text{O}}_{2\text{max}}$ ) to maximum (100% $\dot{\text{V}}{\text{O}}_{2\text{max}}$ ) intensities. They demonstrated that acute intense exercise increased amylin levels in well-trained individuals in a similar fashion to that of insulin. Amylin also remained elevated for much of a 1-hour post-exercise recovery period. Follow-up work by their group determined the effects of prolonged moderate intensity exercise (90 mins of treadmill running 60% of $\dot{\text{V}}{\text{O}}_{2\text{max}}$ ) on amylin and other glucoregulatory hormones. Their results indicated that in a post-prandial state, prolonged exercise induced a decline in insulin, C-peptide, and amylin concentrations to a similar magnitude as a rested (but fed) control arm whilst glucose levels were maintained (42). Thus, the results underscore the role of glucoregulatory endocrine adjustments in sustaining blood glucose concentrations during prolonged exercise, and in amylin as a key contributor of exercise energy balance.

##### Type 1 diabetes

Amylin secretion is diminished (or even absent) in patients with T1D with the degree being generally correlated with the degree of insulin deficiency (43, 44). It has been suggested that this diabetes-induced attenuation in circulating amylin contributes to the susceptibility to severe hypoglycaemia (45). The replacement of amylin alongside insulin could protect against fuel exhaustion through replenishment of hepatic glycogen stores (46). Previous studies have shown that compared to treatment with a) insulin alone and b) insulin plus placebo, co-administration of the amylin analogue pramlintide and insulin using closed loop control leads to a reduction in both the rate and magnitude of increase in post-prandial glucose (47–49). To our knowledge there are no research studies characterising serum amylin responses to exercise in people with T1D. Given its role in mediating glucose, consideration for the use of amylin, amylin antagonists or amylin regulators during exercise in humans with T1D is worthy of exploration.

#### Glucagon (α-cell)

##### Healthy state

Glucagon plays an important role in determining the rate of hepatic glycogenolysis and gluconeogenesis during exercise (50, 51). In healthy man, plasma glucagon concentrations rise with physical exercise in a manner that is dependent on both physical exercise intensity and duration (30). Trained individuals appear to elicit smaller rises in plasma glucagon relative to untrained persons resulting in lower concentrations at the same relative and absolute exercise intensity (30). Brief dynamic exercise can produce small plasma glucagon concentration rises, but this may be due to an artefact of large and rapid blood flow redistribution with the onset of high intensity exercise. Plasma glucagon concentrations may not rise during brief maximal exercise when sympathoadrenal activity is high, but are considerably elevated during prolonged, submaximal exercise where sympathoadrenal activity is moderate (52). Furthermore, the rise in glucagon may last 60 mins or more after exercise; at a time when plasma catecholamine concentrations are markedly reduced. Hence, autonomic nervous system activity may not the sole determinant for the exercise-induced rise in glucagon concentrations (52). Rather, the decline in glucose is postulated as the most influential determinant since glucagon responses can be considerably blunted by glucose ingestion and/or the maintenance of euglycaemia (53). The sensitivity of α-cell to glucagon release due to a small decrease in plasma glucose seems to be heightened by exercise and exacerbated by lowered insulin concentrations (6). It is known that the activation of β-cells generates an inhibitory paracrine signal on α-cells to suppress glucagon secretion supported by the fact that glucagon is higher during exercise in states of low insulin (6).

##### Type 1 diabetes

The observation of attenuated glucagon responses to exercise in T1D vs healthy controls is rather ambiguous in literature with some (12, 54, 55), but not others (56–58), observing the effect. It is worth noting that in the studies that report no statistically significant difference in glucagon responses to exercise between people with vs without T1D (56, 57) the response appears to be consistently numerically lower in those with T1D regardless of variations in exercise and/or cohort characteristics. Even in the absence of differences in the change in glucagon over exercise in those with or without T1D, the ubiquitous reduction in insulin in healthy controls results in a decrease in the insulin: glucagon ratio (6), whilst this is unchanged or even reversed in T1D.

In people with T1D, the plasma glucagon response to exercise is abolished following prolonged bouts of antecedent hypoglycaemia (59, 60). This blunting effect appears to be more pronounced in men than women (100% vs 43% respectively, p<0.05) (60) and carries over into some neuroendocrine (adrenaline, noradrenaline and growth hormone) responses, suggesting a greater reduction in sympathetic drive in men (60). Glucagon responsiveness to exercise is also attenuated by antecedent exposure to physiologic and pharmacologic cortisol elevation without hypoglycaemia in T1D (61), reinforcing α-cell sensitivity to prior stress.

Part of the variance in the magnitude of change in glucagon with exercise may also be due to hepatic extraction. Portal vein glucagon concentrations are approximately three-fold higher than peripheral values during exercise, so do not reflect the physiologically relevant portal vein concentrations (50, 60). Hence, further research is needed to unpack the potential physiological mechanisms driving variations in the glucagon response around exercise and it’s quantification from the portal vein alongside C-peptide/amylin would provide insightful information.

Beyond the endogenous α-cell response, use of exogenous glucagon during exercise is fast becoming an area of notable clinical interest (62). Recent work involving insulin pump users demonstrated superiority of subcutaneous mini-dose glucagon (150 μg) over basal insulin dose reduction (50%) in preventing exercise-induced hypoglycaemia (63). Even in the absence of hypoglycaemia *per se*, pre-exercise administration of subcutaneous glucagon (200 μg) has been shown to preserve normoglycaemia during moderate intensity continuous exercise (45 mins at 50% HR_max_) by eliciting a much smaller drop in plasma glucose than that of a placebo (saline) arm (glucagon: -0.9 ± 2.8 vs saline: −3.1 ± 2.8 mmol.L^-1^, p=0.002) (64). Furthermore, use of a dual-hormone (insulin and glucagon) compared to single-hormone (insulin only) AID system was associated with a complete avoidance of time spent in hypoglycaemia (single hormone: 11% vs dual hormone: 0%, p<0,001) and 100% maintenance of euglycaemia (single hormone: 71% vs dual hormone: 100%, p<0,001) during announced exercise in people with T1D (65). Nevertheless, the commercialisation of these devices is yet to take place.

#### Somatostatin (δ-cells)

##### Healthy man

The somatostatin producing δ-cells are scattered throughout the islet and respond to a multitude of paracrine, endocrine, and neural signals. There are two types of somatostatin: a 14 amino acid form (SST-14) produced primarily by the pancreatic δ-cells and a 28 amino acid form (SST-28) produced by the D-cells of the gastrointestinal tract. The major source of circulating somatostatin is SST-28 whilst the δ-cells are the major source within the islet. Somatostatin is stimulated dose-dependently by glucose in a linear fashion and tonically inhibits the secretion of glucagon and insulin from the neighbouring α- and β-cells (66). This negative feedback mechanism in healthy islets is crucial for optimising glucagon and insulin secretion and hence, glucose regulation. The physiological role of the δ-cells in health and disease has been explored elsewhere (66–68), yet their functional role during exercise is comparatively under-researched.

In healthy man, peripheral plasma concentrations of somatostatin are increased during prolonged periods of acute mild-moderate (40% of $\dot{\text{V}}{\text{O}}_{2\text{max}}$ ) bicycle exercise (pre-exercise: 12.8 ± 1.2 vs post-exercise: 17.7 ± 0.6 pmol.L^-1^) (69). Furthermore, sustained acute exercise (i.e., 90-km cross-country ski race lasting 4.45-6.50 hours) performed under suspected conditions of metabolic catabolism was associated with a considerable increase in plasma somatostatin (pre-exercise: 6.1 ± 0.8 vs post-exercise: 26.9 ± 4.7 pmol.L^-1^, p < 0.001). In this study, post-race oral feeding of 100g glucose in seven of the subjects normalised the plasma concentration within 30 minutes, but the concentration remained elevated in the five subjects who had no post-race caloric supply. These results indicate a close relationship between somatostatin and glucose during caloric deficiency (70). Preventing exercise-induced changes in glucagon and insulin with somatostatin can considerably reduce glucose production *via* attenuating hepatic glycogenolysis, underscoring its influential role in mediating energy availability for working muscles.

##### Type 1 diabetes

Even outside the context of exercise, there is a relative scarcity of information detailing the influence of T1D on somatostatin secretion in isolated human islets. The pathological transition of β-cells from dysfunction to failure is the core event of diabetes. In that sense, the β-cells no longer serve as the source of stimuli for δ-cell activity (68), the result of which can be glucose volatility depending on the under- or over-secretion of somatostatin (68). Experiments in rat models of diabetes are suggestive of impaired counter-regulatory glucagon secretion during insulin-induced hypoglycaemia due to increased somatostatin signalling, the effects of which can be reversed by somatostatin receptor 2 antagonism (SSTR2a) (66, 71–75). Thus, there may be a need to consider the δ-cells as a therapeutic target in T1D, particularly under conditions of hyperinsulinaemic-hypoglycaemia (66); a situation encountered with dynamic physical exercise. In a rodent model, Leclair et al. (76) showed that intraperitoneal administration of PRL-2903, a selective SSTR2 antagonist, enhanced glucagon secretion during exercise/hypoglycaemic challenges (30 minutes of treadmill running @ ~80% $\dot{\text{V}}{\text{O}}_{2\text{max}}$ with end of exercise blood glucose values reaching ≤3.9 mmol.L^-1^) in rats with streptozocin-induced diabetes relative to placebo (154 mmol.L^-1^ NaCI). A subcutaneous bolus of insulin (Humulin R) was injected 60 mins before running to replicate the hyperinsulinaemia/hypoglycaemia commonly observed in humans with T1D undertaking exercise. Glucagon levels failed to rise during exercise with saline but increased three-to-sixfold with PRL-2903 (p<0.05). Furthermore, glucagon responsiveness using SSTR2a was glucose sensitive since glucagon concentrations only rose during hypoglycaemia. Nevertheless, hypoglycaemia still occurred towards the end of exercise with SSTR2a, and the induction of glucagon wavered in the post-exercise period when plasma glucose reached its nadir. The authors postulated these data to be attributable to a product of sustained hyperinsulinaemia, transient elevations in glucagon secretion, and/or low glycogen stores (76). Currently, research in people with T1D is lacking. Nevertheless, in appraising rodent based work, others have highlighted the potential utility of SSTR2 antagonism in mitigating hypoglycaemia around exercise in humans with T1D (77). Even so, given the ubiquitous expression of SSTR2 in several tissues (e.g., the stomach, adrenal medulla, cerebral cortex and hypothalamus), translation to human studies requires initial safety testing (67, 77).

#### Pancreatic polypeptide (γ-cells)

##### Healthy state

Pancreatic polypeptide (PP) has been shown to play a role in glucose homeostasis through its regulation of hepatic insulin receptor gene expression and hepatocyte insulin receptor availability. PP is also a marker of vagal efferent input to the pancreas and can therefore serve as an indicator of parasympathetic nervous system activity during ‘stress’. In healthy man, plasma PP concentrations gradually rise during acute progressive exercise ranging from mild to maximal intensity (78). A more pronounced (5-fold) increase occurs during prolonged (90 mins), mild-moderate intensity exercise and the magnitude of this rise is significantly lowered (2-fold over rest) by short-term (2 months) endurance training (79). Beta-adrenergic stimulation during graded exercise has been shown to augment PP secretion whilst alpha-adrenergic stimulation exerts an inhibitory effect (78). In healthy man, the PP response to a subsequent stress stimulus (hypoglycaemia and exercise) appears to be reduced following prior exposure to episodes of metabolic insult i.e., prolonged hypoglycaemia, elevated cortisol and/or sustained exercise (61, 80–83). Indeed, repeating acute exercise (90 mins cycling at 50% $\dot{\text{V}}{\text{O}}_{2\text{max}}$ ) on the same day is associated with an attenuated PP response, concurrent with lower catecholamine, growth hormone, and cortisol responses (84).

##### Type 1 diabetes

Using an identical study design, albeit as a separate trial, to the works mentioned in healthy man above (i.e., 90 mins cycling at 50% $\dot{\text{V}}{\text{O}}_{2\text{max}}$ ), Galassetti et al. (60), concluded that individuals with T1D have blunted PP responses to exercise compared to healthy controls (60, 85, 86). When retrospectively collapsing averaged data from these studies for the purpose of this review, end of exercise PP responses are proportionately lower in T1D relative to healthy controls whether euglycaemic exercise is performed following previous day antecedent hypoglycaemia (T1D: 40 pmol.L^-1^ vs healthy controls: 45 pmol.L^-1^) or euglycaemia (T1D: 33 pmol.L^-1^ vs healthy controls: 38 pmol.L^-1^). However, when accounting for lower basal PP levels in T1D, relativised exercise-induced increases from baseline appear to be fairly similar (2-3-fold), irrespective of exposure to prior metabolic insult. Nevertheless, direct *intra*-study comparisons of the PP response to exercise in people with vs without T1D are missing, and meaningful interpretation is therefore difficult. With the relative scarcity of data available for PP, studies in which concentrations are characterised in response to exercise of varying intensity, modality, and duration would provide insightful information about its biological role(s) in those with and without T1D.

#### Ghrelin (ϵ-cells)

##### Healthy state

The majority of early ghrelin research was dedicated to its orexigenic effect. However, ghrelin has since been recognised as a key regulator of glucose homeostasis (87). The presence of ghrelin receptors on pancreatic α- and β-cells supports the notion of autocrine/paracrine signalling with ghrelin with physiological relevance. However, the proportionately small (~1%) amount of ghrelin secretion from pancreatic origin is likely to be indistinguishable against the background of circulating concentrations and hence, the majority of information is likely from non-pancreatic sources. Nevertheless, the observed inverse relationship between circulating insulin and ghrelin is well documented in literature and demonstrates inhibitory feedback between the two hormones (88).

Though some ambiguity exists, general consensus from various reviews suggests that acute exercise suppresses acyl ghrelin production in man (89–91). Pooled data from 12 studies involving 118 young, lean, and metabolically healthy males undertaking moderate- to high-intensity aerobic exercise (at intensities ranging from 56% to 83% of $\dot{\text{V}}{\text{O}}_{2\text{peak}}$ ) spanning 30 (n=2), 60 (n=11) and 90 (n=4) minutes in duration showed that circulating acylated ghrelin AUC was 24% lower during exercise compared with rested control (exercise: 99 ± 94 vs control: 131 ± 106 pg.mL^−1^.h^−1^, p<0.001). The exercise-induced suppression of ghrelin carried over for several hours into the recovery period, where there was a 16% decrement in acylated ghrelin AUC in the 3 to 8 hours after exercise cessation (exercise: 108 ± 101 vs rested control: 128 ± 120 pg.mL^−1^.h^−1,^ p=0.024) (91). The mechanistic bases of these observations are unclear but may involve augmented sympathetic output and/or gastric mucosal ischemia resulting from redistribution of blood flow from the splanchnic circulation towards the skeletal muscles during exercise. Even so, certainty in the influence of exercise (acute and chronic) on pancreatic-derived ghrelin is still unclear.

##### Type 1 diabetes

Relative to healthy controls, people with T1D have lower fasting ghrelin concentrations and this depression remains apparent even after insulin therapy initiation (92–95). Furthermore, T1D is associated with abnormalities in ghrelin secretory patterns evidenced by a failure to adequately increase following provision of a mixed meal (93, 95). However, there is a lack of research translating these findings to an exercising context. Given the contributory role of ghrelin in mediating glucose homeostasis and its apparent alteration in response to exercise in healthy man, it is somewhat surprising to learn of the scarcity of research exploring the influence of exercise on ghrelin in people with T1D. Further research is needed to delineate differences, if any, in ghrelin responses to exercise in those with vs without T1D and whether there are any clinically relevant implications on metabolic control.

### Relevance of these findings and concluding remarks

Despite good guidance and recommendations by healthcare professionals based on research findings, the incidence of physical sedentariness in people with T1D is high. Variable glucose responses to the same activity, uncertainty in how best to manage insulin therapy, and the fear of hypoglycaemia prevail as major barriers to regular exercise participation. Finding ways in which we can reduce these burdens is fundamental in helping to encourage regular exercise. A key step in this process is advancing our understanding of the body’s response to exercise and how we can use this information to inform rationale decision making. Given that the main pathophysiology of T1D includes the pancreatic β-cells, an intuitive place to start is by focusing our attention on the organ of origin.

As we continue to move forward with technological and pharmacological developments, it is perhaps prudent to first take a step back and understand the physiological role of the healthy endocrine pancreas during exercise in people with and without diabetes. By broadening our understanding of the factors involved in exercise from a physiological perspective, we stand a greater chance of providing optimal clinical care through informed decision making.

One hope from this review is to raise awareness of the pivotal role of the pancreatic-islet cells in facilitating glucose control during exercise in health and disease. From this review it is clear that whilst our understanding of the contributory roles of the pancreatic alpha and beta cells to glucose homeostasis during exercise is well versed, far less is known about the other islet-cell hormones. Whether similarities or differences in these responses need consideration from a clinical care point of view remains to be established and more research is needed to provide answers.

## Author contributions

OM - Led literature search, draft composure, and draft review. SS and MC - Aided literature search, draft composure, and draft review. SB - Aided draft composure, draft review and provided expert knowledge. KN and RB -Aided literature search, draft composure, draft review and provided expert knowledge. All authors approved the final submitted version.

## Conflict of interest

The authors declare that the research was conducted in the absence of any commercial or financial relationships that could be construed as a potential conflict of interest.

## Publisher’s note

All claims expressed in this article are solely those of the authors and do not necessarily represent those of their affiliated organizations, or those of the publisher, the editors and the reviewers. Any product that may be evaluated in this article, or claim that may be made by its manufacturer, is not guaranteed or endorsed by the publisher.
